# Publisher Correction: In situ light-field imaging of octopus locomotion reveals simplified control

**DOI:** 10.1038/s41586-025-09512-y

**Published:** 2025-09-05

**Authors:** Kakani Katija, Christine L. Huffard, Paul L. D. Roberts, Joost Daniels, Jon Erickson, Denis Klimov, Henry A. Ruhl, Alana D. Sherman

**Affiliations:** Monterey Bay Aquarium Research Institute, Moss Landing, CA USA

**Keywords:** Motility, Biomedical engineering, Marine biology

Correction to: *Nature* 10.1038/s41586-025-09379-z Published online 6 August 2025

This article was originally published under standard Springer Nature license (© The Author(s), under exclusive licence to Springer Nature Limited). It is now available as an open-access paper under a Creative Commons Attribution 4.0 International license, © The Author(s).

