# Supplementary material for: In situ light-field imaging of octopus locomotion reveals simplified control
Source: Nature. 2025 Aug 6;646(8086):865–71. doi: 10.1038/s41586-025-09379-z (PMC12545212; doi:10.1038/s41586-025-09379-z)
Supplement: Supplementary file 1 — Reporting Summary [file 41586_2025_9379_MOESM1_ESM.pdf]

nature portfolio

Corresponding author(s): Kakani Katija

Last updated by author(s): Jun 17, 2025

Reporting Summary

Nature Portfolio wishes to improve the reproducibility of the work that we publish. This form provides structure for consistency and transparency in reporting. For further information on Nature Portfolio policies, see our Editorial Policies and the Editorial Policy Checklist.

Statistics

For all statistical analyses, confirm that the following items are present in the figure legend, table legend, main text, or Methods section.

n/a	Confirmed	
☐	☒	The exact sample size (*n*) for each experimental group/condition, given as a discrete number and unit of measurement
☐	☒	A statement on whether measurements were taken from distinct samples or whether the same sample was measured repeatedly
☒	☐	The statistical test(s) used AND whether they are one- or two-sided<br>*Only common tests should be described solely by name; describe more complex techniques in the Methods section.*
☒	☐	A description of all covariates tested
☒	☐	A description of any assumptions or corrections, such as tests of normality and adjustment for multiple comparisons
☐	☒	A full description of the statistical parameters including central tendency (e.g. means) or other basic estimates (e.g. regression coefficient) AND variation (e.g. standard deviation) or associated estimates of uncertainty (e.g. confidence intervals)
☒	☐	For null hypothesis testing, the test statistic (e.g. F, t, r) with confidence intervals, effect sizes, degrees of freedom and P value noted<br>*Give P values as exact values whenever suitable.*
☒	☐	For Bayesian analysis, information on the choice of priors and Markov chain Monte Carlo settings
☒	☐	For hierarchical and complex designs, identification of the appropriate level for tests and full reporting of outcomes
☒	☐	Estimates of effect sizes (e.g. Cohen's *d*, Pearson's *r*), indicating how they were calculated

Our web collection on statistics for biologists contains articles on many of the points above.

Software and code

Policy information about availability of computer code

Data collection	General instrument control and operation software is provided through open-source repositories reported in the software policy. Proprietary software RxLive 6.0 by Raytrix was used to adjust and save camera sensor information, and derive spatial scales.
Data analysis	RxLive 6.0 was also used to export 3D-reference imagery, which was then analyzed by custom analysis code run in MATLAB R2022b. This code is provided publicly in a GitHub repository.

For manuscripts utilizing custom algorithms or software that are central to the research but not yet described in published literature, software must be made available to editors and reviewers. We strongly encourage code deposition in a community repository (e.g. GitHub). See the Nature Portfolio guidelines for submitting code & software for further information.

Data

Policy information about availability of data

All manuscripts must include a data availability statement. This statement should provide the following information, where applicable:

- Accession codes, unique identifiers, or web links for publicly available datasets
- A description of any restrictions on data availability
- For clinical datasets or third party data, please ensure that the statement adheres to our policy

All image data used for the analysis described in this manuscript, as well as derived, processed data, is provided on Zenodo at 10.5281/zenodo.10795493

Research involving human participants, their data, or biological material

Policy information about studies with human participants or human data. See also policy information about sex, gender (identity/presentation), and sexual orientation and race, ethnicity and racism.

Reporting on sex and gender	Not applicable.
Reporting on race, ethnicity, or other socially relevant groupings	Not applicable.
Population characteristics	Not applicable.
Recruitment	Not applicable.
Ethics oversight	Not applicable.

Note that full information on the approval of the study protocol must also be provided in the manuscript.

Field-specific reporting

Please select the one below that is the best fit for your research. If you are not sure, read the appropriate sections before making your selection.

☐ Life sciences ☐ Behavioural & social sciences ☒ Ecological, evolutionary & environmental sciences

For a reference copy of the document with all sections, see nature.com/documents/nr-reporting-summary-flat.pdf

Ecological, evolutionary & environmental sciences study design

All studies must disclose on these points even when the disclosure is negative.

Study description	Specialized cameras were used to quantify octopus kinematics and locomotion characteristics in-situ at over 3000 meter depth using a remotely operated vehicle (ROV).
Research sample	The research sample consisted of all kinematics data collected of crawling Muusoctopus robustus at the deepwater site at Davidson Seamount in a single dive of a remotely operated vehicle. During the dive, data collection occurred on any animals that were actively crawling upon first observation, and showed minimal if any disturbance behavior upon approach (such as hiding in the substrate). Considerations were given to approach the animal with minimal disturbance to the sea floor and other animal life.
Sampling strategy	Animal sex was determined but not used for decision on inclusion in the research sample. As described above, we collected data on all actively crawling individuals within the time frame of the ROV dive.
Data collection	Data was collected by cameras that recorded the entire ROV dive, as well as our imaging instrument that was manually triggered to record video when the animal of interest was framed well and displayed natural behavior.
Timing and spatial scale	Limited time at depth constrained all data collection within a timeframe of approximately 6 hours, with opportunistic sampling based on observed animal behavior.
Data exclusions	Only data where no 3D data could be extracted was excluded.
Reproducibility	Access limitations precluded us from repeating the sampling on a different day, but behaviors of octopus in archival footage (without 3D measurements) appear to be comparable to those observed in our sample.
Randomization	Due to the large population of octopus at the site, and the natural variation in behaviors, we believe our opportunistic sampling should represent a random sample.
Blinding	No blinding was performed, but sample information such as size and sex was determined from video footage and 3D data after the kinematics analysis had taken place.

Did the study involve field work? ☒ Yes ☐ No

Field work, collection and transport

Field conditions	Deep sea Octopus Gardens located on Davidson Seamount in the Monterey Bay National Marine Sanctuary

Location	35.518911∘N, 71 122.64085∘W; ~ 3230 m deep
Access & import/export	The work was supported by a California Department of Fish and Wildlife license (to KK) and a general use permit for the Monterey Bay National Marine Sanctuary (to MBARI).
Disturbance	When landing the observational vehicle on the seafloor, there is potential for damaging biota. To minimize these disruptions, we would only study animals in areas that were free from other potentially impacted biota.

Reporting for specific materials, systems and methods

We require information from authors about some types of materials, experimental systems and methods used in many studies. Here, indicate whether each material, system or method listed is relevant to your study. If you are not sure if a list item applies to your research, read the appropriate section before selecting a response.

Materials & experimental systems

n/a	Involved in the study
☒	☐ Antibodies
☒	☐ Eukaryotic cell lines
☒	☐ Palaeontology and archaeology
☐	☒ Animals and other organisms
☒	☐ Clinical data
☒	☐ Dual use research of concern
☒	☐ Plants

Methods

n/a	Involved in the study
☒	☐ ChIP-seq
☒	☐ Flow cytometry
☒	☐ MRI-based neuroimaging

Animals and other research organisms

Policy information about studies involving animals; ARRIVE guidelines recommended for reporting animal research, and Sex and Gender in Research

Laboratory animals	No laboratory animals were used in this study.
Wild animals	We studied Muusoctopus robustus (octopuses) in the field and did not collect them for further observations. We could not determine age of individuals.
Reporting on sex	Individuals of both sexes were observed crawling or swimming at the site. The sex of individual octopuses were determined by watching multiple perspectives and identifying the easily-recognizable mating arm of males.
Field-collected samples	No wild animals were used in this study.
Ethics oversight	No ethical approval was required since animals were studied non-invasively in the wild.

Note that full information on the approval of the study protocol must also be provided in the manuscript.

Plants

Seed stocks	Not applicable.
Novel plant genotypes	Not applicable.
Authentication	Not applicable.
